# GCBRGCN: Integration of ceRNA and RGCN to Identify Gastric Cancer Biomarkers

**DOI:** 10.3390/bioengineering12030255

**Published:** 2025-03-03

**Authors:** Peng Zhi, Yue Liu, Chenghui Zhao, Kunlun He

**Affiliations:** 1 Chinese PLA Medical School, Chinese PLA General Hospital, Beijing 100853, China; shengxinzhipeng@163.com; 2 Medical Innovation Research Department of PLA General Hospital, Chinese PLA General Hospital, Beijing 100853, China; 3 Key Laboratory for Research and Evaluationof Artificial Intelligence Medical Devices, Chinese PLA General Hospital, Beijing 100853, China; 4 Medical Engineering Laboratory of Chinese PLA General Hospital, Chinese PLA General Hospital, Beijing 100853, China; 5 Key Laboratory of Biomedical Engineering and Translational Medicine, Ministry of Industry and Information Technology, Chinese PLA General Hospital, Beijing 100853, China; 6 School of Computer Science and Technology, National University of Denfense Technology, Changsha 410073, China; yueliu19990731@163.com

**Keywords:** relational graph convolutional network, gastric cancer, competing endogenous RNA, biomarker

## Abstract

Gastric cancer (GC) is a prevalent malignancy, and the discovery of biomarkers plays a crucial role in the diagnosis and prognosis of GC. However, current strategies for identifying GC biomarkers often focus on a single ribonucleic acid (RNA) class, neglecting the potential for multiple RNA types to collectively serve as biomarkers with improved predictive capabilities. To bridge this gap, our study introduces the GC biomarker relation graph convolution neural network (GCBRGCN) model which integrates the competing endogenous RNA (ceRNA) network with GC clinical informations and whole transcriptomics data, leveraging the relational graph convolutional network (RGCN) to predict GC biomarkers. It demonstrates exceptional performance, surpassing traditional machine learning and graph neural network algorithms with an area under the curve (AUC) of 0.8172 in the task of predicting GC biomarkers. Our study identified three unreported potential novel GC biomarkers: CCNG1, CYP1B1, and CITED2. Moreover, FOXC1 and LINC00324 were characterized as biomarkers with significance in both prognosis and diagnosis. Our work offers a novel framework for GC biomarker identification, highlighting the critical role of multiple types RNA interaction in oncological research.

.

## 1. Introduction

Gastric cancer (GC), a highly prevalent neoplastic disorder, occupies the fifth position in global cancer incidence and the third in cancer-related mortality, with an estimated annual toll of approximately 800,000 fatalities [1]. The discovery and validation of GC biomarkers are of paramount importance for the precise diagnosis and prognostic stratification of patients. Recent advancements in the identification of biomarkers for GC are indeed significant; for instance, the 12-miR analyzer demonstrated a remarkable 87% sensitivity and an exceptional 93.9% specificity within a prospective cohort encompassing 4566 patients [2]. However, the identification of novel biomarkers remains a formidable challenge, attributable to the intrinsic complexity and heterogeneity characterizing GC [3].

With the advent of advanced molecular biology techniques and a more profound comprehension of the mechanisms underlying tumorigenesis, a plethora of methodologies has been developed to identify GC biomarkers. Yang et al. [4] employed Kaplan–Meier and Cox regression analyses to reveal that the over-expression of HAMP could serve as an independent prognostic biomarker for GC patients. Similarly, Azari et al. [5] discovered that elevated expression levels of Mir21, Mir133a, Mir146b, and Mir29c were correlated with higher mortality rates and could potentially serve as early detection biomarkers in early-stage GC patients using the support vector machine (SVM) algorithm. However, these methodologies, which focus on single-class biomarkers, are limited in their ability to capture the intricate interactions among different types of biomarkers. Recent studies have highlighted the potential of competitive endogenous RNA (ceRNA) networks to significantly enhance the predictive power of disease biomarkers [6,7,8]. Recent studies have highlighted the potential of ceRNA networks in significantly enhancing the prediction of disease biomarkers. This approach emphasizes the role of messenger RNAs (mRNAs) and long non-coding RNAs (lncRNAs) in competitively binding microRNAs (miRNAs), forming a complex regulatory network that modulates gene expression through ceRNA interactions. By integrating these complex interactions, it is possible to identify key regulatory RNAs involved in the pathogenesis of GC, offering innovative avenues for biomarker discovery and development.

Furthermore, the advent of artificial intelligence, particularly graph neural networks (GNNs), has heralded a powerful tool for the discovery of network-based biomarkers [9,10,11]. For instance, EMOGI [12] integrates protein–protein interaction (PPI) networks with multi-omics data to identify cancer driver genes. Similarly, MOGONET [13] leverages multi-omics data to identify cancer biomarkers through the application of GNNs. However, these methodologies predominantly utilize homogeneous graphs derived from PPI networks, which limits their applicability to heterogeneous graphs, such as those found in ceRNA networks. In the context of heterogeneous graphs, Gao’s graph autoencoder has demonstrated potential in predicting associations between lncRNA-protein coding gene pairs [14], while Peng et al. [15] constructed three heterogeneous networks to identify cancer driver genes using graph convolution networks. Nevertheless, these methods are constrained in their capacity to uncover multi-RNA type biomarkers, suggesting a need for more sophisticated approaches that can fully exploit the complexity and heterogeneity of biological networks to enhance the discovery of innovative biomarkers.

In this study, we introduce the GC biomarker relation graph convolutional network (GCBRGCN) model, which seamlessly integrates ceRNA networks with clinical informations and whole transcriptomics data specific to GC. Employing the relational graph convolutional network (RGCN), our model is designed to predict GC biomarkers with enhanced accuracy. Our approach innovatively consolidates various RNA types within the biomarker identification process, thereby transcending the limitations inherent in current methodologies when confronting the intricacies of biological networks. We propose a novel and efficacious strategy for the detection of potential biomarkers within the domain of GC research. This strategy not only highlights the potential of multi-RNA type analyses in oncological studies but also underscores the importance of integrating diverse data types to achieve a more comprehensive understanding of the molecular underpinnings of GC.

## 2. Materials and Methods

### 2.1. Overview of GCBRGCN

**Figure 1:**
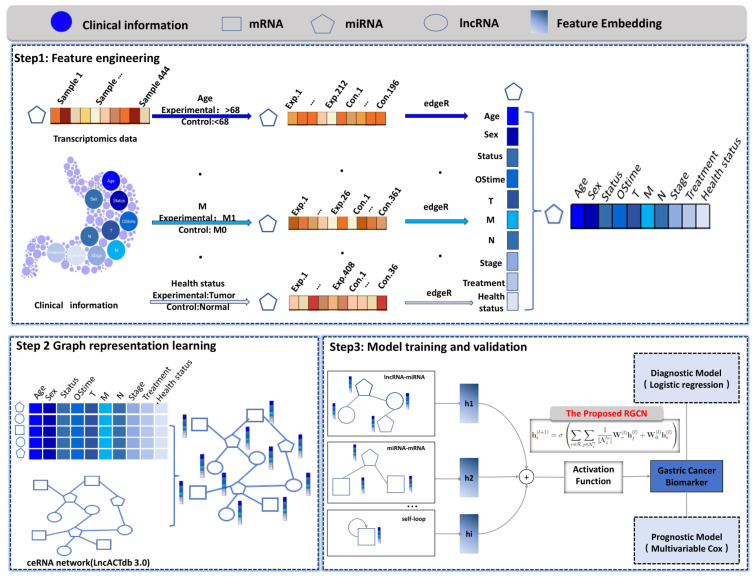
The workflow of the proposed GCBRGCN model. Step 1: Feature Engineering. Clinical data and GC whole transcriptome sequencing data were collected. Samples were stratified based on clinical information, and differential expression analysis was performed using edgeR. The calculated log-fold change values of differentially expressed genes were utilized as node features. Step 2: Graph Representation Learning. Nodes with ten characteristics were embedded and integrated into a ceRNA heterogeneous network, where nodes represent different RNA molecules, including mRNA, lncRNA, and miRNA. Step 3: Model Training and Validation. The RGCN algorithm was employed for node classification, leveraging a multi-layer message passing mechanism to capture higher-order topological features. Based on the classification results, key biomarkers associated with gastric cancer were identified to construct diagnostic and prognostic models, and their potential molecular mechanisms were further investigated.

We introduce a pioneering algorithm named GCRGCN which was founded to synergistically merge ceRNA networks on RGCN with GC whole transcriptomics data and clinical informations. The research workflow, as delineated in Figure 1, encompasses three distinct steps. First, clinical data and GC whole transcriptome sequencing data were collected. To facilitate differential expression analysis, samples were stratified based on clinical characteristics, and the edgeR package was employed to identify log-fold change (logFC) values which were extracted as node features. Next, a ceRNA heterogeneous network was constructed using data from the LncACTdb 3.0 database, where nodes represent different RNA molecules, including mRNA, lncRNA, and miRNA, while edges reflect their regulatory interactions. Each node was assigned a 10-dimensional feature vector, incorporating clinical attributes. Subsequently, the RGCN algorithm was applied for node classification, leveraging a multi-layer message passing mechanism to extract higher-order topological features. Based on the classification results, novel biomarkers associated with GC were identified and subsequently used to construct a diagnostic model using logistic regression and a prognostic model using multivariable Cox regression.

### 2.2. Data Collection and Preprocessing

#### 2.2.1. Construction of the ceRNA Heterogeneous Graph

The ceRNA network used in this study was derived from the LncACTdb 3.0 database [16], which provides experimentally validated interactions among ceRNAs. LncACTdb 3.0 is a comprehensive and well-curated resource offering high-quality data on interactions between mRNAs, miRNAs, and lncRNAs, with a particular focus on their roles in cancer-related regulatory networks. By leveraging this database, we obtained a ceRNA network consisting of 497 nodes, including 163 mRANAs, 151 miRNAs, and 183 lncRNAs, connected to each other by 357 edges. We ensured that the ceRNA network employed in our analysis is biologically relevant and supported by robust experimental evidence, providing a solid foundation for our graph-based approach to biomarker discovery in GC.

#### 2.2.2. Biomarker Annotation for Nodes

Nodes within the ceRNA network were annotated as potential GC biomarkers based on curated information from several well-established databases, including Cancer Gene Census (CGC) [17], BBCancer [18], IntOGen [19], LncRNA-Disease [20], and GeneCard [21]. The CGC (https://cancer.sanger.ac.uk/census/, accessed on 1 January 2020) provides a curated catalog of genes with somatic mutations implicated in GC, which helped identify potential biomarkers. The BBCancer (http://bbcancer.renlab.org/download, accessed on 1 January 2020) integrates cancer gene and biomarker information to annotate nodes based on their association with GC. IntOGen (https://www.intogen.org/, accessed on 1 January 2020), a comprehensive compendium of somatic mutations across cancers, provided insights into cancer-associated genes used for labeling relevant nodes. The LncRNA-Disease database (http://www.rnanut.net/lncrnadisease/, accessed on 1 January 2020) links lncRNAs to diseases, including GC, and was used to annotate lncRNA nodes. Finally, GeneCard (https://www.genecards.org/, accessed on 1 January 2020), a comprehensive human gene database, provides functional annotations for genes involved in cancer and helped label genes in the ceRNA network with potential relevance to GC. Nodes associated with GC were marked as 1, while those not associated with GC were marked as 0.

#### 2.2.3. Transcriptomics Feature Generation

We extracted miRNA-seq, RNA-seq data and clinical information for GC from The Cancer Genome Atlas (TCGA), a comprehensive and widely used resource providing multi-dimensional genomic data for GC. TCGA offers high-quality data, including gene expression, clinical outcomes, mutation profiles, and methylation information, enabling a deeper understanding of cancer biology. These datasets were preprocessed to ensure consistency and quality, including normalization and the removal of low-quality samples. After matching and filtering, we retained only those samples containing both clinical information and both miRNA-seq and RNA-seq data, resulting in a final dataset of 408 GC patients and 36 normal controls. Following the approach outlined in EMOGI [12], we calculated node features based on relative gene expression changes rather than absolute values, with gene expression logFC determined by stratifying the samples according to ten key clinical factors: (1) Age: divided at 68 years, with younger patients as the experimental group and older as the control. (2) Sex: males as the experimental group and females as the control. (3) Survival status: deceased patients as the experimental group and survivors as the control. (4) Overall survival time (OStime): less than 426 days as the experimental group and more than 426 days as the control. (5) Cancer staging: advanced stages (III and IV) characterized by locoregional invasion and distant metastasis as the experimental group and early stages (I and II) as the control. (6) Tumor classification (T): T3 and T4 indicating tumors that have penetrated the serosa or invaded adjacent structures as the experimental group and T1 and T2 tumors as the control. (7) Regional lymph node involvement (N): N1-N3 as the experimental group and N0 as the control. (8) Metastasis (M) Status: M1 (distant metastasis) as the experimental group and M0 (no distant metastasis) as the control. (9) Treatment modalities: radiation therapy as the experimental group and pharmaceutical therapy as the control. (10) Health status: tumor as the experimental group and normal as the control. These classifications generated ten unique features for each node, allowing for an in-depth assessment of how different clinical factors affect gene expression profiles. A comprehensive description of these features is provided in Table 1.

We constructed the adjacency matrix for model training based on the ceRNA network. For each node within the network, a 10-dimensional feature vector was derived by integrating clinical grouping information with GC transcriptomic data. Subsequently, node labels were assigned using known GC biomarkers from databases such as CGC and BBCancer, thereby constructing the expression matrix for model input.

#### 2.2.4. Model Training

We aim to embed the nodes in the graph into the latent space and classify them into two classes, i.e., biomarker and non-biomarker. We adopt the GNNs to model the nodes in the graphs. Previous graph neural networks follow the simple differentiable message-passing framework. It is formulated as follows:

(1) $$h_{i}^{\left(l+1\right)}=\sigma \left(\sum_{m\in M_{i}}F_{m}\left(h_{i}^{\left(l\right)},h_{j}^{\left(l\right)}\right)\right),$$

where $h_{i}^{\left(l\right)}\in R^{d\left(l\right)}$ denotes the latent code of the *i*-th node in the *l*-th layer of the neural network. Additionally, $d^{\left(l\right)}$ denotes the latent dimension number of the *l*-th layer of the neural network. *m* denotes the message information, and $M_{i}$ is the message information set of the *i*-th node. $F_{m}$ denotes the aggregation function for the incoming message information. σ is the activation function, such as ReLU, Sigmoid function, etc. In the classical graph convolutional network (GCN), the incoming messages come from the node neighbors. Neighborhood messages are embedded via a simple linear transformation, i.e., $W^{\left(l\right)}h_{j}^{\left(l\right)}$, where $W^{\left(l\right)}$ denotes the transformation matrix of the *l*-th layer. Note that all the messages adopt the same transformation. To be specific, the encoding process of the GCN is formulated as follows.

(2) $$H^{l+1}=\sigma \left({\tilde{D}}^{-1/2}\tilde{A}{\tilde{D}}^{-1/2}W^{\left(l\right)}H^{l}\right),$$

(3) $$\tilde{A}=A+I,$$

where σ denotes the activation function, $W^{\left(l\right)}$ denotes the transformation matrix at the *l*-layer, and $\tilde{D}$ denotes the degree matrix. Note that the GCN models all the relationships between nodes with the same transformation matrix W, limiting the representation capability. Additionally, GAT introduces the attention mechanism to improve the modeling of the relationship. It is formulated as follows:

(4) $$h_{i}^{\left(l+1\right)}=\sigma \left(\sum_{j\in M_{i}}\alpha_{ij}W^{\left(l\right)}h_{j}^{\left(l\right)}\right),$$

where $\alpha_{ij}$ is the attention coefficient. It is calculated as follows:

(5) $$\alpha_{ij}=\frac{\exp(\mathrm{LeakyReLU}(a(W^{l}h_{i}∥∥W^{l}h_{j})))}{\sum_{k\in M_{i}}\exp(\mathrm{LeakyReLU}(a(W^{l}h_{i}∥∥W^{l}h_{k})))},$$

where *a* denotes a single-layer feed-forward neural network. However, GAT still cannot capture the different types of relationships.

In this paper, to better model the various relationships between nodes with different types, we adopt the RGCN as the encoder. The message-passing patter is formulated as follows:

(6) $$h_{i}^{\left(l+1\right)}=\sigma \left(\sum_{r\in R}\sum_{j\in N_{i}^{r}}\frac{1}{|N_{i}^{r}|}W_{r}^{\left(l\right)}h_{j}^{\left(l\right)}+W_{0}^{\left(l\right)}h_{i}^{\left(l\right)}\right),$$

where $h_{i}^{\left(l+1\right)}$ denotes the the *i*-th node embedding in the (l+1)-th neural network layer. $W_{r}^{\left(l\right)}$ denotes the transformation matrix for the relation *r* in the *l*-th layer. Additionally, $W_{0}^{\left(l\right)}$ denotes the transformation matrix for the self-message in the *l*-th layer. In addition, $N_{i}^{r}$ denotes the neighborhood set of the *i*-th node with the relation *r*, and $|N_{i}^{r}|$ is the element number of the set. Moreover, R is the relationship set, and r∈R is one relationship in all relationships. Different from the simple GCN model, RGCN introduces the relation-specific transformation. Namely, $W_{r}^{\left(l\right)}$ will embed the different relationships into the different latent spaces. Additionally, the self-connection is considered as a special relation for each entity in the graph, and $W_{0}^{\left(l\right)}$ aims to embed them into the latent space. In this manner, we embed the nodes in the graph into latent space and obtain the node embedding $H\in R^{N\times d}$, where *N* denotes the number of the nodes and *d* denotes the dimension number of latent features. Then, we conduct an entity classification task on learned node embeddings. Specifically, cross-entropy loss is minimized as follows:

(7) $$L=-\sum_{i=1}^{N}\sum_{k=1}^{K}Y_{ik}\ln{\left(\mathrm{softmax}\left(H\right)\right)}_{ik},$$

where $H\in R^{N\times d}$ denotes the node embeddings from the R-GCN. *N* and *K* denote the number of the nodes and the number of the classes, respectively. With the activation function softmax, the network outputs the probability of each nodes to the classes.

#### 2.2.5. Parameter Configuration in Model Training

Striking a balance between convergence velocity and model performance was crucial, which we addressed through the meticulous hyperparameter optimization of parameters: dropout rate, self-loops, learning rate, embedding dimension, number of hidden layers, and epoch. We set the dropout rate to 0.6 (Table A1), enabled self-loops to be set to TRUE (Table A2), established a learning rate of 0.1 (Table A3), chose an embedding dimension of 100, structured the model with three hidden layers, and determined the epoch count to be 100. This parameter tuning aimed to enhance model performance, ensuring both swift convergence and strong generalization capabilities, especially when dealing with heterogeneous graph-structured data.

### 2.3. Performance Benchmarking of Different Methods

To establish a benchmark for comparison, we integrated six established algorithms into our study. First, the heterogeneous graph attention network (HAN) [22] is a sophisticated algorithm that extends traditional graph convolutional networks by incorporating attention mechanisms, allowing for the fine-grained capture of complex interactions among various node and edge types in heterogeneous graphs. Second, the GCN [23] adapts convolutional operations to graph-structured data, enabling learning and prediction from graph data. Third, the graph attention network (GAT) [24] employs an attention mechanism within GNNs to dynamically learn node relationships, thereby enhancing model performance and interpretability. Additionally, we also compare K-nearest neighbor (KNN), random forest (RF), and eXtreme Gradient Boosting (XGBoost) algorithms, which are widely used in the field of machine learning.

### 2.4. Model Evaluation

The dataset was split into training and testing sets with a 7:3 ratio. We utilized key metrics, including accuracy (ACC), recall, precision, and the F1-score. Additionally, we employed the receiver operating characteristic (ROC) curve to assess model effectiveness. The ROC curve is derived by plotting the true-positive rate (TPR) against the false-positive rate (FPR) across a range of thresholds, providing a visual representation of the model’s ability to distinguish between positive and negative classes. We also adopted the area under the ROC curve (AUC) as a quantitative measure of model performance, offering a comprehensive assessment of the model’s predictive power across all classification thresholds.

(8) $$\mathrm{ACC}=\frac{TP+TN}{TP+TN+FP+FN}$$

(9) $$\mathrm{Recall}=\frac{TP}{TP+FN}$$

(10) $$\mathrm{Precision}=\frac{TP}{TP+FP}$$

(11) $$F1-\mathrm{score}=\frac{2\times Precision\times Recall}{Precision+Recall}$$

The results of evaluation indicators can be divided into true positive (TP), false positive (FP), true negative (TN) and false negative (FN).

To evaluate the stability and reliability of the models, a five-fold cross-validation approach was employed. In this process, the dataset was randomly partitioned into five subsets of equal size. For each fold, the model was trained on four subsets and validated on the remaining subset. This procedure was repeated five times, ensuring that each subset was used exactly once as the validation set while the remaining subsets were utilized for training. To facilitate a comprehensive comparison of the models, the mean and standard deviation of the performance metrics were calculated across the five folds for each model. The mean value represents the average performance of the model, while the standard deviation quantifies the variability in performance across the folds, providing insights into the model’s consistency. Models exhibiting lower standard deviations are considered to demonstrate more stable performance, whereas those with higher standard deviations indicate greater variability, which may compromise their generalizability. The mean and standard deviation for each model were computed as follows:

(12) $$\mu =\frac{1}{N}\sum_{i=1}^{N}x_{i}$$

(13) $$\sigma =\sqrt{\frac{1}{N}\sum_{i=1}^{N}{\left(x_{i}-\mu \right)}^{2}}$$

where *N* is the number of folds (i.e., N=5), and $x_{i}$ represents the performance metric (e.g., ACC, precision, recall, etc.) for the *i*-th fold.

### 2.5. Novel GC Biomarkers’ Assessment

Genes identified as biomarkers by the GCBRGCN model, but not previously annotated in the aforementioned cancer gene databases, were deemed novel GC biomarkers. To elucidate the biological functions and pathways associated with the novel biomarkers, we performed Kyoto Encyclopedia of Genes and Genomes (KEGG) and Gene Ontology (GO) enrichment analyses using the clusterProfiler package. The KEGG pathway enrichment analysis was conducted to identify potential signaling pathways related to gastric cancer. The novel biomarkers were annotated against the KEGG database, and pathways with a corrected *p* < 0.05 were considered statistically significant. For GO enrichment analysis, the novel biomarkers were mapped to GO terms across three categories: biological process (BP), cellular component (CC), and molecular function (MF). Terms with a *p* < 0.05 were considered significantly enriched. The enrichment results were visualized using ggplot2 to comprehensively illustrate the functional annotations and pathway interactions of the novel biomarkers. To visualize the regulatory network of the novel biomarkers, we constructed and analyzed the interaction network using Cytoscape (version 3.10.2). In the network, nodes represent novel biomarkers and their regulatory molecules, while edges represent the interactions between them.

#### 2.5.1. Diagnostic Validation

To ascertain whether novel GC biomarkers are viable for diagnosis, we initially employed the edgeR algorithm to identify differentially expressed genes between GC patients and healthy individuals. The criteria were set at $\left|\mathrm{logFC}\right|>1$ and a false discovery rate (FDR) <0.05. Next, we determined the intersection between the differentially expressed genes and the novel GC biomarkers, yielding a set of intersecting genes. The pROC package was then utilized to calculate the ROC curves for these intersecting genes and derive the AUC. Novel GC Biomarkers with AUC values exceeding 0.6 were considered novel diagnostic biomarkers for GC. Biomarkers with AUC>0.8 and AUC>0.7 were selected to construct diagnostic models using Logistic regression. Logistic regression allows for the modeling of binary outcomes and is commonly applied in biomarker-based diagnostic prediction. In addition to utilizing internal validation datasets, we procured external validation datasets (GSE184336) from the Gene Expression Omnibus database to bolster the reliability of our findings.

#### 2.5.2. Prognostic Validation

We implemented a stringent approach to confirm the prognostic predictive capacity of the newly identified GC biomarkers. Initially, we developed a multivariable Cox regression model, incorporating patients’ survival data, vital status, and expression levels of the novel GC biomarkers to determine their prognostic predictive value. Subsequently, using the standardized expression levels of these biomarkers along with their regression coefficients, we derived risk scores for individual patients, categorizing them into high- and low-risk groups for GC. Following this, we executed Kaplan–Meier survival analysis to assess survival probabilities over time. Additionally, we conducted univariate Cox regression analysis to reaffirm the prognostic significance of these biomarkers. The univariate Cox regression analysis allows for the evaluation of individual biomarkers’ contribution to survival risk before considering the effects of other variables. Finally, we formulated a nomogram that integrates the prognostic model with clinical information, streamlining its application in clinical settings. All analyses were conducted using R version 4.3.0.

## 3. Results

### 3.1. Performance Comparison of GCBRGCN and Existing Methods in Biomarker Discovery of GC

**Figure 2:**
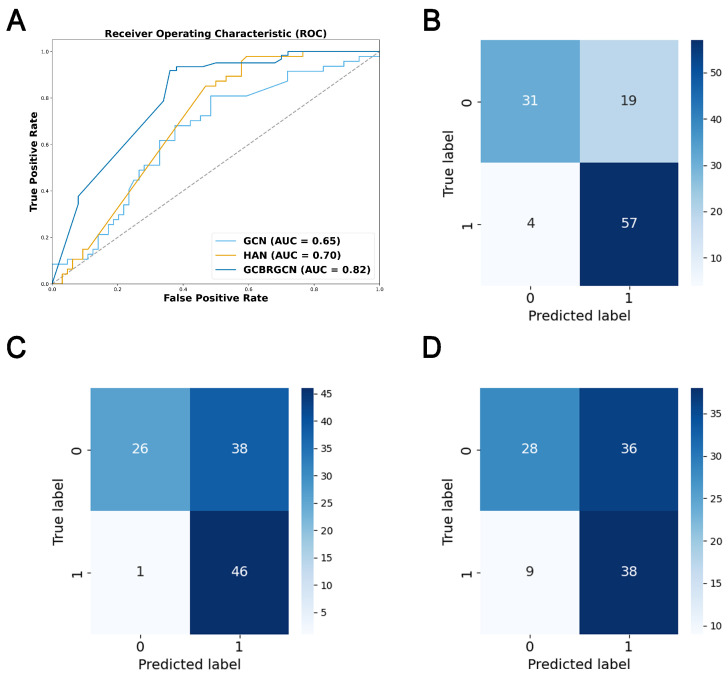
ROC curve and confusion matrix for model performance evaluation. (**A**) ROC curves of GCBRGCN, HAN and GCN models. (**B**) Confusion matrix of the GCBRGCN model. (**C**) Confusion matrix of the HAN model. (**D**) Confusion matrix of the GCN model.

The model was trained on the training set and evaluated on the testing set, with the performance metrics reported in Table 2. When compared to other GNN models, like HAN, GCN, and GAT, GCBRGCN achieved the best results in terms of accuracy (0.7928), precision (0.7500), F1-score (0.8321), and AUC (0.8172), using the same network configurations and node attribute settings (Figure 2). Although the HAN model achieved the highest recall (0.9787), GCBRGCN’s recall (0.9344) was only slightly lower. In comparison with conventional machine learning techniques, such as KNN, RF, and XGBoost, GCBRGCN demonstrated superior performance, highlighting the advantages of GNN algorithms in GC biomarker identification through the integration of graph structures. Further, the five-fold cross-validation results show that GCBRGCN performs well in accuracy (0.798 ± 0.093), precision (0.7419 ± 0.1434), recall (0.8147 ± 0.2265), F1-score (0.8214 ± 0.149), and AUC (0.7358 ± 0.0932) metrics and exhibits stable performance among different folds. The performance is stable between folds with a small standard deviation, demonstrating its strong robustness (Table 3, Table A4, Table A5, Table A6, Table A7, Table A8, Table A9 and Table A10).

### 3.2. Ablation Experiments

**Figure 3:**
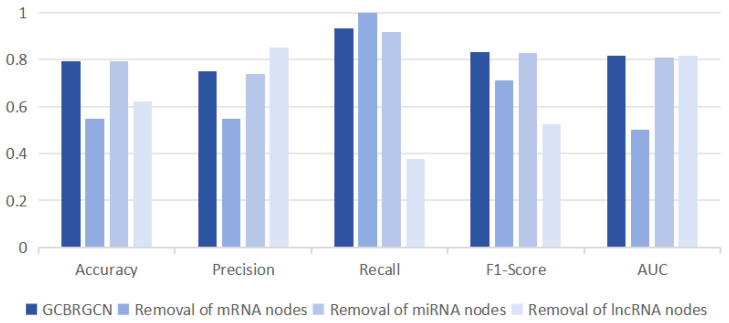
Comparison of classification results of removing one type of node at a time based on GCBRGCN model.

To assess the individual contributions of mRNA, miRNA, and lncRNA to the GCBRGCN model, we conducted ablation studies, systematically removing one type of node at a time. Consistent with our expectations, we observed that the removal of mRNA nodes had the most significant impact on model performance, resulting in the largest reductions in accuracy (0.2432), precision (0.2005), and AUC (0.3172). This outcome could be attributed to mRNAs being the primary functional RNAs, and the fact that the majority of known GC biomarkers are mRNA-based. LncRNAs had the most substantial impact on the model’s recall (0.5574), and they also significantly affected the accuracy (0.1712) and F1-score (0.3094). Interestingly, miRNAs had a negligible effect on model performance across all metrics, including precision (0.0132), recall (0.0164), F1-score (0.0025), and AUC (0.0090), possibly owing to the inherent stability of miRNA biomarkers (Figure 3).

### 3.3. Biological Interpretation of Novel GC Biomarkers

**Figure 4:**
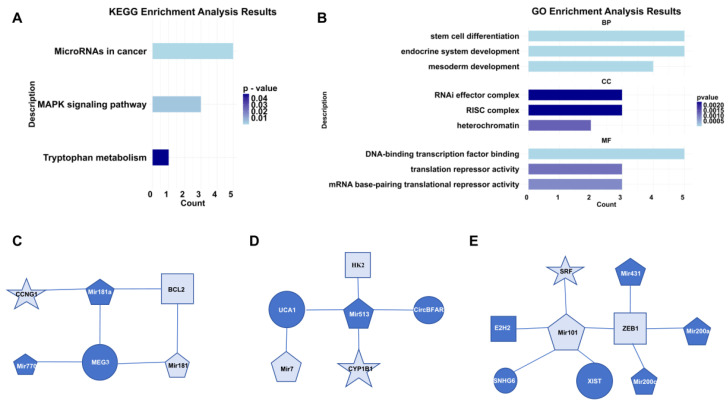
Biological interpretation of novel GC biomarkers. (**A**) Results of KEGG pathway enrichment analysis. (**B**) Results of GO enrichment analysis. (**C**) Subnetwork of CCNG1. (**D**) Subnetwork of CYP1B1. (**E**) Subnetwork of SRF. In these networks, distinct shapes represent different types of RNAs, where the size of each shape corresponds to the degree of connectivity, and dark blue coloring indicates RNAs that are known biomarkers.

Our analysis revealed that novel GC biomarkers were predominantly associated with miRNAs in cancer, the MAPK signaling pathway, and tryptophan metabolism according to KEGG enrichment analysis (Figure 4A). Additionally, they were enriched in endocrine system development, heterochromatin, and DNA-binding transcription factor binding based on GO enrichment analysis (Figure 4B). To elucidate the interactions between novel GC biomarkers and established ones, we constructed three subnetwork focusing on the novel GC biomarkers. Our analysis revealed that the novel GC biomarkers CCNG1, BCL2, and Mir181 were linked to MEG3 and Mir181a which were known as GC biomarkers. MEG3 has been shown to suppress the proliferation and invasion of GC cells in vitro, and it can upregulate BCL2 through its compete combine against Mir181a [25] (Figure 4C). Similarly, the novel GC biomarkers HK2, Mir7, and CYP1B1 were connected with the known GC biomarkers UCA1, Mir513, and circBFAR. The UCA1/Mir513/CYP1B1 axis plays a role in modulating cisplatin resistance in human GC cells [26], and circBFAR has been demonstrated to enhance GC proliferation by targeting the Mir/HK2 axis [27]. Mir7 can bind to specific sites on UCA1, modulating the target EGFR [28] (Figure 4D). Additionally, the novel GC biomarkers Mir101 and ZEB1 emerged as central nodes in the subnetwork. Mir101 can directly target and repress the expression of the novel GC biomarker SRF [29]. Oncogene SNHG6 could regulate the Mir101/ZEB1 axis at the post-transcriptional level and recruit the enhancer of EZH2 to the promoter of P27, thereby silencing P27 expression at the transcriptional level [30] (Figure 4E).

#### 3.3.1. Novel Diagnostic Biomarkers

**Figure 5:**
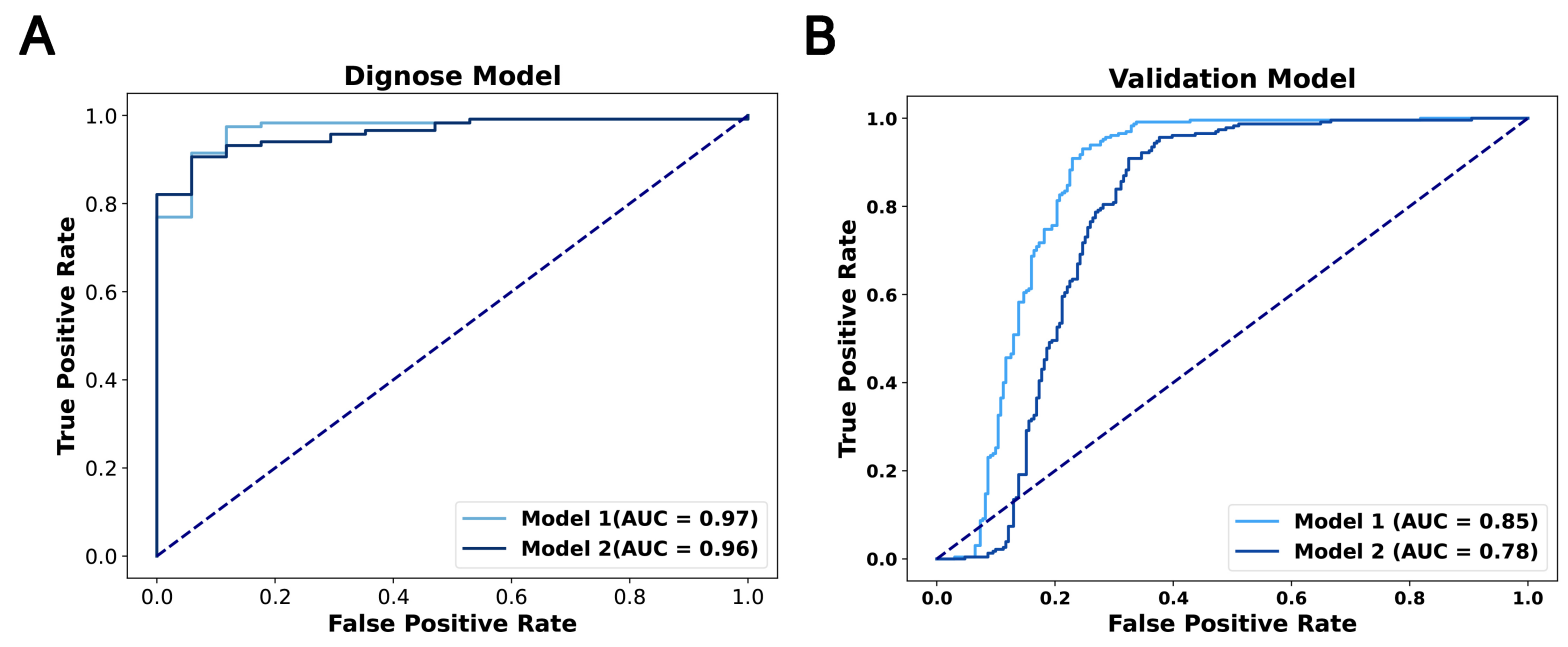
(**A**) ROC curves for the diagnosis model using TCGA datasets. (**B**) ROC curves for the diagnosis model using external validation datasets.

**Figure A1:**
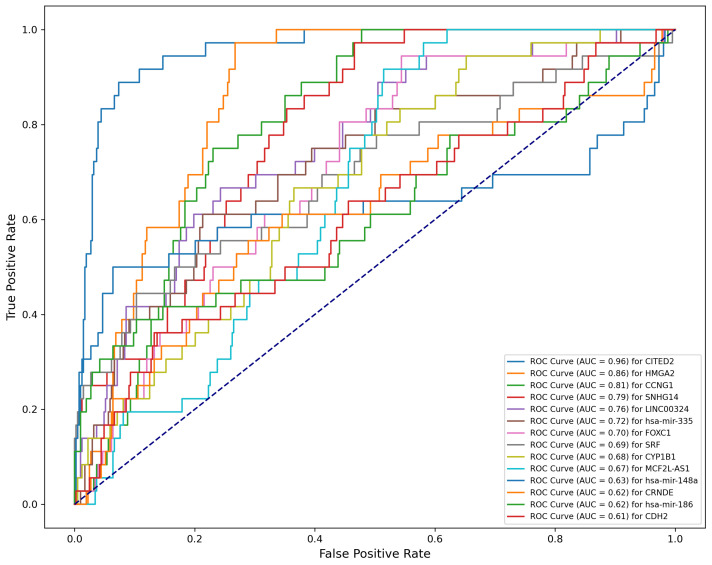
Performance of new predictive diagnosis biomarkers (AUC>0.6).

We discovered 14 novel diagnostic biomarkers with an AUC greater than 0.6 (Figure A1). From these, we developed diagnostic model one, which included seven biomarkers with an AUC above 0.7: CITED2 (0.96), HMGA2 (0.86), CCNG1 (0.81), SNHG14 (0.79), LINC00324 (0.76), Mir335 (0.72) and FOXC1 (0.7). This model exhibited an AUC of 0.97 in the TCGA datasets, and the external validation datasets reported an AUC of 0.85. To streamline the model, we then created a second diagnostic model, utilizing only three of the biomarkers: CITED2, HMGA2 and CCNG1. This simplified model achieved an AUC of 0.96 in TCGA datasets, with the external validation datasets indicating an AUC of 0.78, demonstrating significant predictive AUC (Figure 5A,B). The performance of these diagnostic models confirms the efficacy of the GCBRGCN in identifying diagnostic prediction biomarkers.

#### 3.3.2. Prognostic Biomarkers

**Figure 6:**
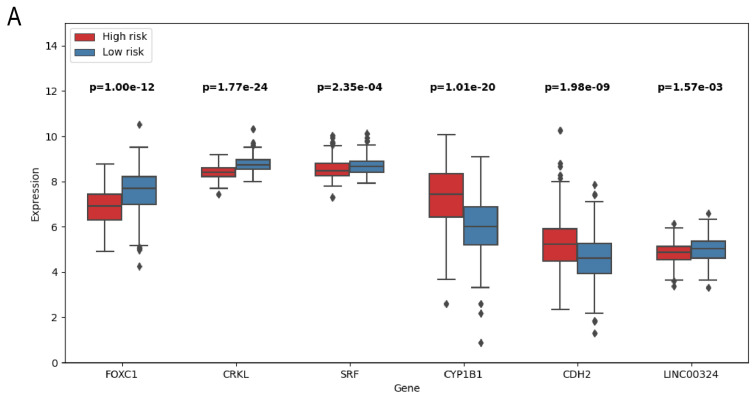

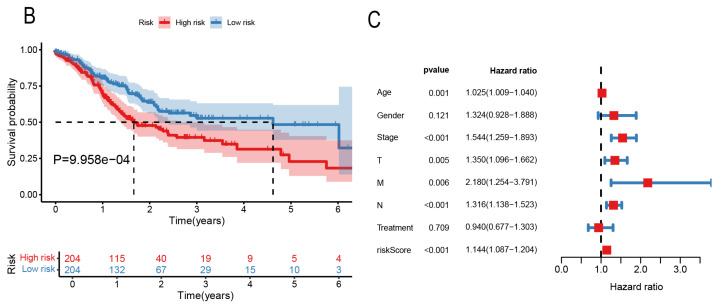
(**A**) Boxplot of prognostic related genes in high- and low-risk groups. (**B**) Kaplan–Meier survival curves of patients in high- and low-risk groups. (**C**) Forest map of bivariate Cox risk regression analysis of forest map.

**Figure 7:**
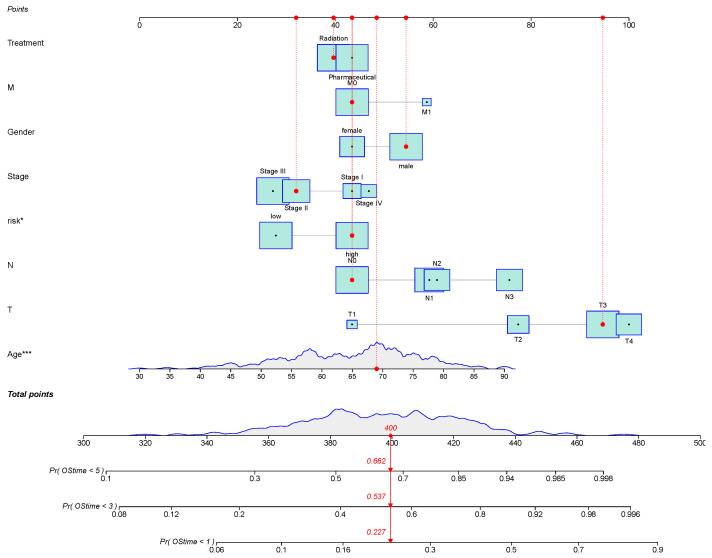
Nomogram to predict the probability of 1-year, 3-year, or 5-year OS in patients with GC. Predictor points are obtained from the points scale according to the prognostic contribution of each variable subset. These, added together, give a total point score which can be translated into probability of survival at a specific timepoint (1-year, 3-year, or 5-year) by charting the score on the total points scale and projecting onto the probability of survival scale. The survival estimates are given as the probability of survival. For instance, if a patient’s total score is 400 points, calculated based on actual clinical information, the corresponding probabilities of OStime being less than 1 year, 3 years, and 5 years are 0.227, 0.537, and 0.662, respectively. Statistical significance of variables is indicated with asterisks, reflecting the strength of the association between the variable and OStime. * means *p* < 0.05; *** means *p* < 0.001.

Six novel prognostic biomarkers, including LINC00324, FOXC1, CRKL, SRF, CYP1B1 and CDH2, were assessed via multivariate Cox analysis. The samples were divided into a high-risk group and low-risk group by calculating the risk score, and the prognostic model was constructed. All prognostic biomarkers showed significant differential expression between the high- and low-risk groups (Figure 6A). Through Kaplan–Meier survival, we found that the prognosis of the high-risk group was significantly worse than that of the low-risk group (P = 9.958 $\times \;10^{-4}$; Figure 6B). Furthermore, univariate Cox regression analysis demonstrated that the risk score, constructed based on six prognostic biomarkers, could serve as an independent prognostic indicator for GC patients, with a hazard ratio of 1.144 (95% CI: 1.087–1.204; Figure 6C). Furthermore, we developed a nomogram to assist clinicians in estimating the 1-year, 3-year, and 5-year survival rates for individual GC patients. For instance, if a patient’s total score is 400 points, calculated based on actual clinical information, the corresponding probabilities of OStime being less than 1 year, 3 years, and 5 years are 0.227, 0.537, and 0.662, respectively (Figure 7).

## 4. Discussion

The significance of different RNA-type biomarkers in GC diagnostics and prognostics is garnering increasing attention within the scientific community. However, the development of comprehensive, multi-molecular biomarker prediction models in this field remains a less-researched area. Addressing this research gap, our study introduces the GCBRGCN model, which has shown superior performance in the discovery of novel GC biomarkers, outperforming both heterogeneous and traditional GNNs as well as conventional machine learning algorithms.

The GCBRGCN model constructs a network based on ceRNA interactions and incorporates critical transcriptomic features alongside pertinent clinical informations, including tumor staging and survival duration. By harnessing the complexity of these interactions through sophisticated heterogeneous GNN analysis, GCBRGCN is capable of uncovering potential novel biomarkers and deciphering their biological significance. This approach offers novel insights into the identification of new GC diagnostic and prognostic biomarkers.

We have identified Mir335, CCNG1, HMGA2, SNHG14, and CITED2 as potential new diagnostic biomarkers for GC. Mir335, recognized as a tumor suppressor gene in GC, is significantly down-regulated in GC compared to normal gastric tissue [31,32]. Furthermore, CCNG1, which is up-regulated in various tumor tissues [33,34], has not yet been identified as a GC diagnosis biomarker. However, our analysis revealed that CCNG1 is regulated by Mir181a and MEG3, which are well-established GC biomarkers [35]. Notably, the expression of MEG3 is decreased in GC patients, and it can up-regulate BCL2 by competitively binding to the Mir181a family, thereby inhibiting the onset of GC [36,37]. Additionally, it has been reported that high levels of HMGA2 are significantly correlated with lymphatic vessel invasion, perineural invasion, and TNM stage [38]. In another study, Dai et al. demonstrated that elevated levels of SNHG14, confirmed by sequencing and qRT-PCR, enhance GC proliferation, invasion, and migration, as demonstrated in vitro and in animal models [39]. Lastly, resistance to anthracycline chemotherapy drugs can be overcome by reactivating the epigenetically silenced CITED2 gene, thus providing a new strategy for GC treatment to enhance chemotherapy sensitivity and drug reactivity [40].

SRF, CRKL, CYP1B1 and CDH2 were identified as new potential prognostic biomarkers for GC. Firstly, SRF, a downstream target of the MAPK/ERK signaling pathway, promotes cell proliferation and the development of GC metastasis [41]. Additionally, SRF fosters the proliferation and invasion of GC cells by suppressing the expression of HOTAIR [29]. Secondly, CRKL, a substrate of BCR-ABL tyrosine kinase, is involved in the transformation process of BCR-ABL into fibroblasts [42]. Mir335 targets CRKL, thereby inhibiting the migration, invasion, and proliferation of tumor cells, arresting the cell cycle at the G0/G1 phase, and promoting apoptosis in GC cells [43]. Thirdly, CYP1B1, a member of the cytochrome P450 supergene family, plays a role in the regulation of several crucial transcription factors through the oxidation and metabolism of various carcinogenic precursors and anticancer drugs [26]. Finally, MFGE8 induces the expression of SNHG14, which in turn promotes the cellular epithelial–mesenchymal transition by stabilizing CDH2 [39].

Notably, FOXC1 and LINC00324 could serve as both prognostic and diagnostic biomarkers. Jiang’s research has shown that increased FOXC1 expression in GC patients is associated with a poor prognosis. At the molecular level, FOXC1 promotes the nuclear translocation of unphosphorylated catenin, which then upregulates c-MYC expression, thereby driving GC cell proliferation [44]. Additionally, Zou discovered that the expression level of LINC00324 is significantly higher in GC tissues than in corresponding normal tissues. The over-expression of LINC00324 correlates with an advanced TNM stage, larger tumor size, lymph node metastasis, and a poor prognosis [45].

It is important to recognize that our study does have certain limitations. Firstly, the integration of multiple RNA molecules and clinical information introduces complexities that may affect the performance of our model. Moreover, the discrepancies between whole transcriptomics data and cancer parameters present challenges in sourcing external test datasets to validate our model, thereby constraining its generalizability.

## 5. Conclusions

In conclusion, the GCBRGCN provides a novel and interpretable method for predicting GC biomarkers by integrating whole transcriptomics data and clinical information with ceRNA networks. Our study identified three unreported potential novel GC biomarkers: CCNG1, CYP1B1, and CITED2. Moreover, FOXC1 and LINC00324 were characterized as biomarkers that were significant in both prognosis and diagnosis. This innovative approach is expected to enhance diagnostic and prognostic capabilities in GC and offers a new paradigm for biomarker discovery in other cancers.

## Figures and Tables

**Table 1:** Node characteristic information.

Clinical Information	Experimental	Control	Unknown
Age	212	196	0
Sex	264	144	0
Status	162	246	0
OStime	203	205	0
Stage	207	177	24
T	292	108	8
N	266	123	19
M	26	361	21
Treatment	202	206	0
Health status	408	36	0

**Table 2:** Performance comparison of GCBRGCN and existing methods in biomarker discovery of GC.

Model	Accuracy	Precision	Recall	F1-Score	AUC
GCBRGCN	0.79	0.75	0.93	0.83	0.82
HAN	0.65	0.55	0.98	0.70	0.69
GCN	0.59	0.51	0.81	0.63	0.62
GAT	0.51	0.56	0.83	0.58	0.54
KNN	0.64	0.66	0.61	0.63	0.70
RF	0.62	0.67	0.53	0.59	0.69
XGBoost	0.64	0.66	0.61	0.63	0.66

**Table 3:** Model performance comparison by five-fold cross-validation.

Model	Accuracy	Precision	Recall	F1-score	AUC
GCBRGCN	0.80±0.09	0.74±0.14	0.81±0.23	0.82±0.15	0.74±0.09
HAN	0.59±0.11	0.55±0.16	0.79±0.23	0.68±0.18	0.69±0.05
GCN	0.56±0.14	0.53±0.18	0.75±0.22	0.59±0.17	0.63±0.03
GAT	0.58±0.17	0.47±0.21	0.85±0.34	0.55±0.22	0.65±0.03
KNN	0.54±0.09	0.50±0.07	0.78±0.13	0.51±0.06	0.61±0.07
RF	0.59±0.06	0.54±0.09	0.65±0.19	0.60±0.08	0.67±0.06
XGBoost	0.59±0.04	0.52±0.07	0.70±0.22	0.59±0.08	0.66±0.04

## Data Availability

The original data and code presented in this study are openly available in https://github.com/PengZhi1906/GCBRGCN, accessed on 1 January 2020.

## Abbreviations

The following abbreviations are used in this manuscript:

GC	Gastric cancer
RNA	Ribonucleic acid
ceRNA	Rompeting endogenous RNA
RGCN	Relational graph convolutional network
GCBRGCN	GC biomarker RGCN
AUC	Area under the curve
PPI	Protein–protein interaction
lncRNA	Long non-coding RNA
miRNA	MicroRNA
mRNA	Messenger RNA
SVM	Support vector machine
GNNs	Graph neural networks
logFC	Log-fold change
CGC	Cancer Gene Census
TCGA	The Cancer Genome Atlas
OStime	Overall survival time
T	Tumor classification
N	Lymph node involvement
M	Metastasis
GNN	Graph convolutional network
HAN	Heterogeneous graph attention network
KNN	K-nearest neighbor
RF	Random forest
XGBoost	eXtreme Gradient Boosting
ACC	Accuracy
ROC	Receiver operating characteristic
TPR	True-positive rate
FPR	False-positive rate
TP	True Positive
FP	False Positive
TN	True Negative
FN	False Negative
KEGG	Kyoto Encyclopedia of Genes and Genomes
GO	Gene Ontology
BP	Biological process
CC	Cellular component
MF	Molecular function
FDR	False discovery rate

## Appendix A

**Table A1:** Dropout in hyperparameter.

Dropout	Accuracy	Precision	Recall	F1-Score	AUC
0	0.7838	0.7402	0.9344	0.8261	0.8169
0.2	0.7838	0.7402	0.9344	0.8261	0.8169
0.4	0.7838	0.7402	0.9344	0.8261	0.8169
0.6	0.7928	0.7500	0.9344	0.8321	0.8172
0.8	0.7928	0.7500	0.9344	0.8321	0.8172

**Table A2:** Self-loop in hyperparameter.

Self-Loop	Accuracy	Precision	Recall	F1-Score	AUC
TRUE	0.7928	0.7500	0.9344	0.8321	0.8172
FALSE	0.7838	0.7403	0.9344	0.8261	0.8169

**Table A3:** Learning rate in hyperparameter.

Learning Rate	Accuracy	Precision	Recall	F1-Score	AUC
0.1	0.7928	0.7568	0.9180	0.8296	0.8182
0.01	0.7928	0.7500	0.9344	0.8321	0.8172
0.001	0.6757	0.6316	0.9836	0.7692	0.8166
0.0001	0.5495	0.5495	1.0000	0.7093	0.3251
0.00001	0.5495	0.5495	1.0000	0.7093	0.3251

**Table A4:** Five-fold cross-validation result of GCBRGCN.

Fold	Accuracy	Precision	Recall	F1-Score	AUC
Fold 1	0.8182	0.5833	0.6364	0.6087	0.7532
Fold 2	0.7778	0.7755	1.0000	0.8736	0.5217
Fold 3	0.7980	0.7419	0.9200	0.8214	0.7967
Fold 4	0.5612	0.3273	0.7500	0.4557	0.6250
Fold 5	0.5714	0.4324	1.0000	0.6038	0.6818

**Table A5:** Five-fold cross-validation result of HAN.

Fold	Accuracy	Precision	Recall	F1-Score	AUC
Fold 1	0.3535	0.25	0.9545	0.3962	0.5924
Fold 2	0.7778	0.7755	1	0.8736	0.5375
Fold 3	0.6768	0.6154	0.96	0.75	0.7714
Fold 4	0.4592	0.3067	0.9583	0.4646	0.761
Fold 5	0.5306	0.4103	1	0.5818	0.7282

**Table A6:** Five-fold cross-validation result of GCN.

Fold	Accuracy	Precision	Recall	F1-Score	AUC
Fold 1	0.6465	0.2593	0.3182	0.2857	0.6057
Fold 2	0.7677	0.7789	0.9737	0.8655	0.6773
Fold 3	0.5253	0.5155	1.0000	0.6803	0.6592
Fold 4	0.2449	0.2449	1.0000	0.3934	0.6118
Fold 5	0.3469	0.3333	1.0000	0.5000	0.5959

**Table A7:** Five-fold cross-validation result of GAT.

Fold	Accuracy	Precision	Recall	F1-Score	AUC
Fold 1	0.737374	0.333333	0.181818	0.235294	0.64876
Fold 2	0.787879	0.808989	0.947368	0.872727	0.629291
Fold 3	0.606061	0.563218	0.98	0.715328	0.665714
Fold 4	0.418367	0.291139	0.958333	0.446602	0.70045
Fold 5	0.367347	0.336957	0.96875	0.5	0.617424

**Table A8:** Five-fold cross-validation result of KNN.

Fold	Accuracy	Precision	Recall	F1-Score	AUC
Fold 1	0.5593	0.5200	0.4815	0.5000	0.6053
Fold 2	0.5763	0.5385	0.5185	0.5283	0.6007
Fold 3	0.5424	0.5000	0.5556	0.5263	0.6319
Fold 4	0.5593	0.5185	0.5185	0.5185	0.6238
Fold 5	0.5517	0.5000	0.4231	0.4583	0.5877

**Table A9:** Five-fold cross-validation result of random forest.

Fold	Accuracy	Precision	Recall	F1-Score	AUC
Fold 1	0.6271	0.5926	0.5926	0.5926	0.6250
Fold 2	0.6102	0.6111	0.4074	0.4889	0.6753
Fold 3	0.6102	0.6000	0.4444	0.5106	0.6470
Fold 4	0.5593	0.5556	0.1852	0.2778	0.5972
Fold 5	0.6034	0.5714	0.4615	0.5106	0.5397

**Table A10:** Five-fold cross-validation result of XGBoost.

Fold	Accuracy	Precision	Recall	F1-Score	AUC
Fold 1	0.5932	0.5517	0.5926	0.5714	0.6175
Fold 2	0.6441	0.6364	0.5185	0.5714	0.6377
Fold 3	0.5932	0.5652	0.4815	0.5200	0.5914
Fold 4	0.5593	0.5217	0.4444	0.4800	0.6053
Fold 5	0.5345	0.4800	0.4615	0.4706	0.5337
